# Integrin‐Targeted, Short Interfering RNA Nanocomplexes for Neuroblastoma Tumor‐Specific Delivery Achieve *MYCN* Silencing with Improved Survival

**DOI:** 10.1002/adfm.202104843

**Published:** 2021-06-30

**Authors:** Aristides D. Tagalakis, Vignesh Jayarajan, Ruhina Maeshima, Kin H. Ho, Farhatullah Syed, Lin‐Ping Wu, Ahmad M. Aldossary, Mustafa M. Munye, Talisa Mistry, Olumide Kayode Ogunbiyi, Arturo Sala, Joseph F. Standing, Seyed M. Moghimi, Andrew W. Stoker, Stephen L. Hart

**Affiliations:** ^1^ Department of Genetics and Genomic Medicine UCL Great Ormond Street Institute of Child Health University College London 30 Guilford Street London WC1N 1EH UK; ^2^ Department of Inflammation Infection and Immunity UCL Great Ormond Street Institute of Child Health University College London 30 Guilford Street London WC1N 1EH UK; ^3^ Centre for Pharmaceutical Nanotechnology and Nanotoxicology Faculty of Health and Medical Sciences University of Copenhagen Universitetsparken 2 Copenhagen 2100 Denmark; ^4^ Department of Histopathology Great Ormond Street Hospital for Children NHS Foundation Trust London WC1N 3JH UK; ^5^ Department of Life Sciences Brunel University London Kingston Lane Middlesex UB8 3PH UK; ^6^ Department of Developmental Biology and Cancer UCL Great Ormond Street Institute of Child Health University College London 30 Guilford Street London WC1N 1EH UK; ^7^ Present address: Department of Biology Edge Hill University Ormskirk L39 4QP UK; ^8^ Present address: National Center for Biotechnology King Abdulaziz City for Science and Technology Riyadh 11442 Saudi Arabia; ^9^ Present address: Cell and Gene Therapy Catapult 12th Floor Tower Wing, Guy's Hospital, Great Maze Pond London SE1 9RT UK; ^10^ Present address: Guangzhou institute of Biomedicine and Health Chinese Academy of Sciences Guangzhou 510530 People's Republic of China; ^11^ Present address: School of Pharmacy, and Translational and Clinical Research Institute, the Faculty of Medical Sciences Newcastle University Newcastle upon Tyne NE1 7RU UK; ^12^ Present address: Colorado Center for Nanomedicine and Nanosafety, Skaggs School of Pharmacy and Pharmaceutical Sciences University of Colorado Anschutz Medical Campus Aurora CO 80045 USA

**Keywords:** MYCN, neuroblastomas, siRNA, tumor‐specific delivery, tumors

## Abstract

The authors aim to develop siRNA therapeutics for cancer that can be administered systemically to target tumors and retard their growth. The efficacy of systemic delivery of siRNA to tumors with nanoparticles based on lipids or polymers is often compromised by their rapid clearance from the circulation by the liver. Here, multifunctional cationic and anionic siRNA nanoparticle formulations are described, termed receptor‐targeted nanocomplexes (RTNs), that comprise peptides for siRNA packaging into nanoparticles and receptor‐mediated cell uptake, together with lipids that confer nanoparticles with stealth properties to enhance stability in the circulation, and fusogenic properties to enhance endosomal release within the cell. Intravenous administration of RTNs in mice leads to predominant accumulation in xenograft tumors, with very little detected in the liver, lung, or spleen. Although non‐targeted RTNs also enter the tumor, cell uptake appears to be RGD peptide‐dependent indicating integrin‐mediated uptake. RTNs with siRNA against *MYCN* (a member of the Myc family of transcription factors) in mice with *MYCN*‐amplified neuroblastoma tumors show significant retardation of xenograft tumor growth and enhanced survival. This study shows that RTN formulations can achieve specific tumor‐targeting, with minimal clearance by the liver and so enable delivery of tumor‐targeted siRNA therapeutics.

## Graphical abstract

This paper describes a new lipid and peptide nanoparticle formulation with potential as a treatment for neuroblastoma that targets tumors specifically by intravenous administration. The nanoparticle delivers a siRNA molecule that silences the proto‐oncogene MYCN as a neuroblastoma tumor‐specific target. The nanoparticle accumulates specifically in tumors in mice and the treatment leads to retarded tumor growth in most cases.

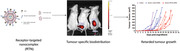

## 1 Introduction

Neuroblastoma is the most common solid tumor of childhood, accounting for 8–10% of all childhood cancers and 15% of cancer‐related deaths in children.^[^ ^1^ ^]^ The current treatment for neuroblastoma patients with severe disease is chemotherapy, followed by surgical resection or radiotherapy.^[^ ^2^ ^]^ Those diagnosed over 1 year of age have a less favorable outlook and often fail to respond even to these aggressive combinations of therapy while others frequently relapse, and so alternative treatments are needed.

Gene amplification of *MYCN* in neuroblastoma is associated with a particularly poor prognosis and occurs in about 20% of cases.^[^ ^3^ ^]^ *MYCN* encodes N‐Myc, a member of the Myc family of transcription factors, controlling expression of genes involved in proliferation, cell growth, protein synthesis, metabolism, apoptosis, and differentiation.^[^ ^4^ ^]^ N‐Myc also interacts with apoptotic factors such as BCL2^[^ ^5^ ^]^ or p53 and may inhibit apoptosis while, conversely, over‐expression of N‐Myc may also drive apoptosis.^[^ ^6^ ^]^ *MYCN* amplification leads to over‐expression of N‐myc, although, interestingly, there are cases where protein levels are increased in tumors without genome amplification.^[^ ^7^ ^]^ *MYCN* expression occurs normally at fetal stages of neurogenic tissue development with little or none expressed in adult tissues and so neuroblastoma‐associated *MYCN* expression is highly tumor specific.^[^ ^8^ ^]^ We and others have demonstrated therapeutic effects of short interfering RNA (siRNA)‐mediated *MYCN* silencing, including cell differentiation or apoptosis, in neuroblastoma models in vitro and in vivo with as little as 50% reduction of *MYCN* mRNA.^[^ ^4^, ^9^ ^]^ *MYCN* is, thus, an attractive therapeutic target for neuroblastoma associated with *MYCN* amplification or N‐myc over‐expression, as it offers a safe, tumor‐specific target in *MYCN*‐amplified tumors.^[^ ^9^, ^10^ ^]^

We propose that a tumor‐specific, *MYCN*‐targeted siRNA (siMYCN) treatment for disseminated neuroblastoma may be achieved by systemic administration of the siRNA encapsulated in a suitable nanoparticle formulation. Neuroblastoma tumors are highly vascularized with a leaky endothelium that may enable extravasation of nanoparticles into the tumor from the circulation,^[^ ^11^ ^]^ or by endothelial transcytosis.^[^ ^12^ ^]^ We have previously described a nanocomplex formulation that enabled tumor‐specific plasmid DNA (pDNA) transfection, with very little transfection of liver or other organs^[^ ^13^ ^]^ and demonstrated pDNA‐expressed cytokine adjuvant immunotherapy;^[^ ^13b^ ^]^ but siRNA therapeutics offer alternative targets and strategies in cancer therapies, such as ectopically expressed oncogenes.

Receptor‐targeted nanocomplexes (RTNs) comprise a formulation of integrin‐targeting peptides and lipids which, on mixing with nucleic acids at optimized ratios form self‐assembling complexes.^[^ ^13^, ^14^ ^]^ The peptide contains a cationic, sixteen‐lysine domain for nucleic acid packaging, and a short cyclic peptide ligand, GACRGDCLG, which binds to integrin cell surface receptors.^[^ ^15^ ^]^ Integrins are highly expressed on tumor cells and exhibit non‐polarized membrane distribution instead of the basolateral distribution seen in healthy tissues, which increases integrin accessibility to targeting ligands, such as RGD peptides.^[^ ^13^, ^16^ ^]^ The RTN lipid components may be cationically or anionically charged, affecting the surface charge of the resulting nanocomplexes^[^ ^14f^ ^]^ while PEGylated liposomes are widely used to shield charge, and confer stealth properties.^[^ ^14e^ ^]^ The neutral, fusogenic dioleoylphosphoethanolamine is essential to mediate endosomal release of the nucleic acids.^[^ ^17^ ^]^ Thus, RTNs contain multiple functionalities to overcome cellular and extracellular barriers to tumor‐targeted transfection and have achieved pDNA transfection of tumors of more than 90% in a murine syngeneic model of neuroblastoma.^[^ ^13^ ^]^

Our aim in this study was to develop novel RTNs for siMYCN delivery to neuroblastoma tumors and to evaluate their in vivo biodistribution, therapeutic effects, and toxicity. We have evaluated and compared the targeted delivery and therapeutic potential of cationic and anionic‐PEGylated formulations and anionics offer the prospects of reduced toxicity in vivo and greater cell‐targeting specificity.^[^ ^14f^ ^]^ RTNs were assessed for systemic delivery of siMYCN formulations to neuroblastoma tumor xenografts and we describe a pharmacokinetic/pharmacodynamic model of the effects of treatment on tumor growth.

## 2 Results and Discussion

### 2.1 Biophysical Characterization of Nanocomplexes

**Figure 1:**
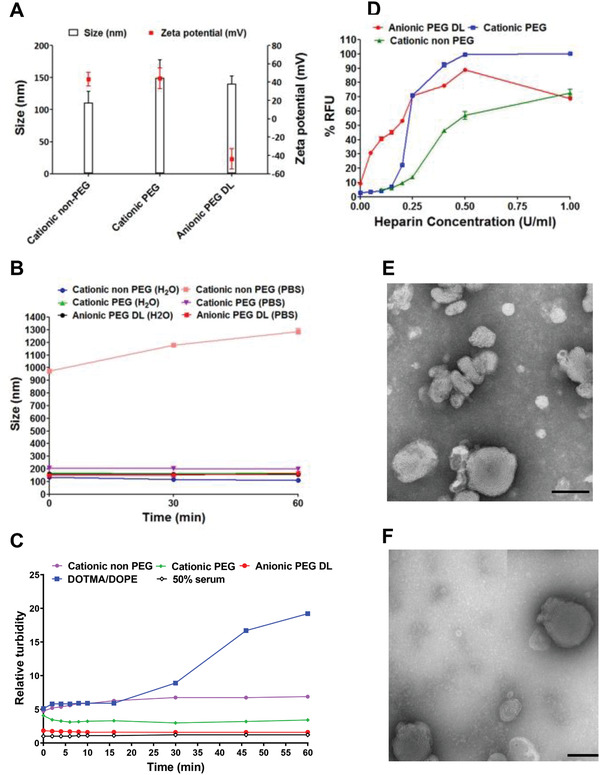
Biophysical characteristics of cationic non‐PEG, cationic‐PEG, and anionic‐PEG nanocomplexes. A) Nanoparticle size and surface charge was measured by dynamic light scattering. B) Size measurements of nanocomplexes over a 1‐h period with nanocomplexes made in either water or PBS. C) The effect of 50% mouse serum concentration on the relative turbidity of cationic, cationic‐PEG, and anionic‐PEG nanocomplexes over a 60 min incubation period. A cationic liposome (DOTMA/DOPE) was used as a positive control of the assay. D) The dissociation properties of cationic and anionic‐PEGylated nanocomplexes were investigated from PicoGreen fluorescence of nanocomplexes, after incubation with heparin (0–1 U mL^−1^), expressed as a percentage of relative fluorescence units (RFU) relative to free siRNA. Negative staining TEM was used to visualize, E) anionic PEG and F) cationic‐PEG nanocomplexes. Scale bar = 100 nm. DL= double‐layered.

The size, charge and stability of siRNA lipid/peptide RTNs were first determined to assess their suitability for in vivo use. The sizes of cationic, cationic‐PEG, and anionic‐PEG siRNA nanocomplexes were 110.7 ± 17.7, 149.4 ± 28.4, and 140.6 ± 11.4 nm, respectively, with corresponding zeta potentials of +43.1 ± 7.4, +44.1 ± 11.2, and −43.7 ± 11.1 mV (**Figure** **1**A). The polydispersity index (PDI) measurements of cationic, cationic‐PEG, and anionic‐PEG siRNA nanocomplexes were 0.402 ± 0.028, 0.344 ± 0.157, and 0.188 ± 0.027, respectively. Each nanocomplex formulation was stable in water while non‐PEGylated cationic nanocomplexes aggregated rapidly in PBS to ≈1290 nm (Figure 1B). The short PEG lipids (PEG_2000_) and their low density in the RTN formulation were selected to confer particle stability in physiological solutions without excessively compromising their transfection efficiency. PEGylated cationic and anionic nanocomplexes were stable in PBS at ≈205 nm and ≈147 nm, respectively (Figure 1B), demonstrating the anticipated shielding effect of this PEGylation strategy.^[^ ^13^, ^18^ ^]^

An alternative sizing modality, nanoparticle tracking analysis (NTA) (Table S1, Supporting Information), showed cationic‐PEGylated formulations with a size of 144 nm, very similar to the zetasizer estimation of 149 nm, although the anionic‐PEG RTNs were somewhat larger by NTA at 243 nm compared to 140 nm by zetasizer analysis. These discrepancies presumably arise from sample polydispersity, since the average hydrodynamic diameters obtained by DLS is a measure of total scattered light from an ensemble of particles, whereas in NTA the measurements are based on the Brownian motion through video analysis and tracking the movement of individual particles. Negative‐staining, transmission electron microscopy (TEM) indicated that anionic‐PEG and cationic‐PEG nanocomplexes were both mainly spherical with sizes consistent with light scattering data (Figure 1E,F).

In a serum‐stability assay, performed as described previously,^[^ ^19^ ^]^ the turbidity of the cationic‐PEG nanocomplexes in mouse serum (50%) was approximately half that of the cationic‐non PEG counterpart, whereas the anionic‐PEG nanocomplexes showed the least aggregation (Figure 1C), due to the steric barrier of PEG in minimizing interaction with serum proteins.^[^ ^14e^ ^]^ On the basis of these observations, PEGylated cationic and anionic formulations were selected for in vivo use.^[^ ^19^ ^]^

The siRNA packaging efficiency of PEGylated nanocomplexes and their dissociation potential in response to heparin was then assessed. While extracellular stability is an essential requirement for an effective siRNA delivery formulation, transfection efficiency is also dependent on release of siRNA within the cell following internalization.^[^ ^20^ ^]^ Nanocomplex dissociation in the presence of heparin reflects their potential to release siRNA in the cytoplasm where high concentrations of anions such as nucleic acids, and sialic acid residues, will be encountered.^[^ ^14^, ^21^ ^]^ PicoGreen‐labeled siRNA was formulated into nanocomplexes to assess packaging efficiency. Cationic PEGylated and non‐PEGylated formulations displayed similar levels of PicoGreen fluorescence quenching in the absence of heparin, while that of anionic formulations was slightly less quenched, although greater than 90%, indicating that all formulations encapsulated the siRNA effectively (Figure 1D). PEGylation appeared to reduce the stability of the cationic nanocomplexes to heparin, while anionic formulations were the least stable, indicating the possibility of easier release of siRNA from the nanocomplex.

### 2.2 In Vitro Transfection Efficiencies

Receptor‐mediated uptake of nanoparticles enhances uptake into targeted cells while minimizing non‐specific delivery.^[^ ^22^ ^]^ Integrins are heterodimeric receptors normally involved in cell–cell and cell–extracellular matrix interactions. In tumor cells, the normal polarized, basolateral display of integrins breaks down, making them more accessible to targeting ligands thus offering tumor‐specific targets.^[^ ^16b^ ^]^ In this study, we used peptide ME27 (K_16_RVRRGACRGDCLG), containing the conserved integrin‐targeting motif Arg‐Gly‐Asp (RGD) motif in a cyclic form, which targets α_v_β_3_, α_v_β_5_, and α_5_β_1_ integrins. This motif is followed by a K_16_ domain for electrostatic siRNA packaging, and an RVRR domain, which is cleavable by the endosomal enzymes cathepsin B and furin. We have shown previously that cleavable peptide linkers enhance intracellular nanocomplex disassembly, and so enhance transfection efficiency.^[^ ^13^, ^23^ ^]^

**Figure 2:**
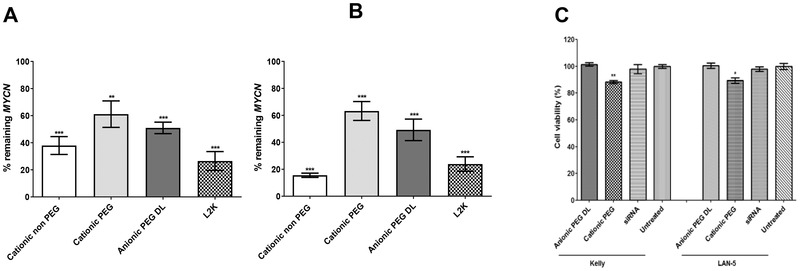
siRNA‐mediated silencing of *MYCN* with nanocomplexes in different cell lines. Cationic non‐PEG, cationic‐PEG, and anionic‐PEG complexes were used in transfections with MYCN siRNA in A) Kelly cells and B) LAN‐5 cells. Nanocomplexes with non‐targeting control siRNA were used in control transfections and L2K/siRNA was used as a positive control while untreated cells received no siRNA. qRT‐PCR assays were normalized to non‐targeting control siRNA formulations. C) Viability of Kelly and LAN‐5 cells following transfection for 24 h with different nanocomplexes at 100 nm siRNA. Viability values were normalized to the untransfected control cells. All transfections were performed in groups of six and mean values were calculated. Asterisks indicate comparisons of specific formulations to the control untransfected cells with statistical significance (*p < 0.05; **p < 0.01). DL= double‐layered.

An optimized siRNA, siMYCN‐3, was selected from preliminary *MYCN* silencing transfections (Figure S1A,B, Supporting Information) then silencing efficiency of *MYCN* in Kelly and LAN‐5 cells was assessed by qRT‐PCR and compared with different RTN formulations. Cationic non‐PEGylated formulations achieved the highest levels of silencing at >60% and >80% in both cell lines (**Figures** **2**A and 2B, respectively). We have shown elsewhere that silencing of *MYCN* at this level reduces protein on western blots and induces apoptosis in Kelly cells and differentiation in another neuroblastoma cell line, IMR32.^[^ ^9b^ ^]]^ While PEGylation slightly reduced silencing efficiency in vitro, we focused primarily on PEGylated formulations for in vivo delivery due to their stability and superior resistance to aggregation compared with non‐PEGylated formulations. Cell viability following transfections with control siRNA showed that the cationic‐PEG nanocomplexes resulted in slightly increased cytotoxicity in both Kelly and LAN‐5 cells (Figure 2C), although viability remained at about 90% in both cell lines, while the anionic‐PEGylated nanocomplexes did not induce any apparent cytotoxicity. These results are consistent with previous reports of the cytotoxicity of cationic liposomes.^[^ ^24^ ^]^

### 2.3 Complement Activation Assay, Cell Viability, and Cellular Effects

**Figure 3:**
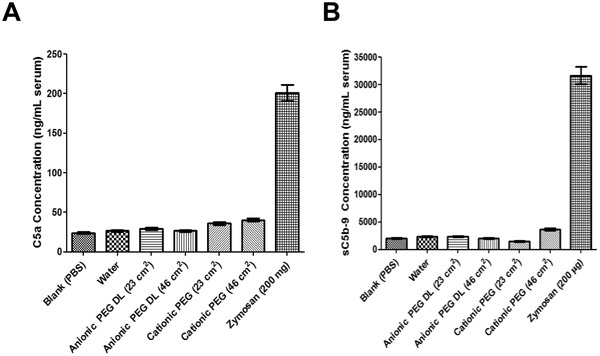
Complement activation assays. A) Quantification of complement activation product of A) C5a and, B) SC5b‐9, in human serum after incubation of nanocomplexes with equivalent total surface areas (23 cm^2^ mL^−1^ serum and 46 cm^2^ mL^−1^ serum, cationic‐PEG and anionic PEG nanocomplexes, respectively), calculated from the NTA data (Table S1 and Figure S2, Supporting Information). Blank (PBS) and positive control Zymosan (200 µg ml^−1^), were tested during the experiment, as was water. DL = double‐layered.

The complement system has an important role in both innate and cognate immunity recognizing danger signals through pattern recognition and clearance by neutrophils, monocytes, and tissue macrophages.^[^ ^25^ ^]^ However, uncontrolled complement activation may elicit acute adverse reactions and promote tumor growth.^[^ ^26^ ^]^ To assess complement activation with RTN formulations, we incubated human serum with cationic‐PEG and anionic‐PEG formulations at comparable surface areas (23 and 46 cm^2^), as complement activation is surface area‐dependent. Surface areas were determined from average size measurements of each sample by the NTA (Table S1 and Figure S2, Supporting Information). The particle concentration values imply a siRNA molecular content per particle of ≈3000 for anionic‐PEG and ≈5000 for cationic PEG nanoparticles. Measurement of the complement‐activation products, sC5b‐9 and C5a showed a slight increase in serum C5a levels for the cationic‐PEG formulation (*p* < 0.05) and anionic‐PEG formulation (*p* < 0.01) compared with water and PBS controls (**Figure** **3**A), while sC5b‐9 remained at background levels (Figure 3B). Zymosan was used as a positive control for complement activation and induced high levels of C5a and sC5b‐9 products (201.3 ± 10.1 ng mL^−1^ C5a, and 31 678 ± 1 583 ng mL^−1^ sC5b‐9, respectively). Thus, cationic‐PEG and anionic‐PEG RTN formulations displayed negligible levels of complement activation, which contrasts with the PEGylated liposome product, Doxil, which activated sC5b‐9 at much higher levels (8560 ± 108.1 ng mL^−1^).^[^ ^27^ ^]^

**Figure 4:**
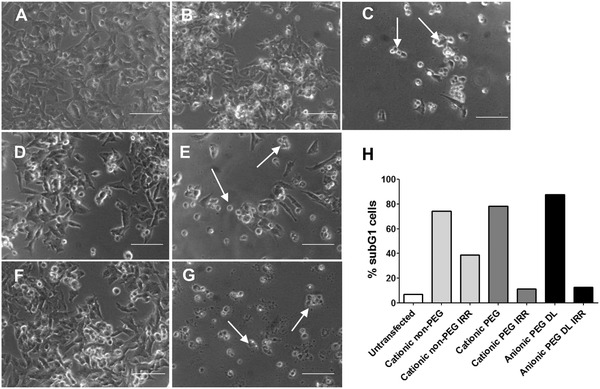
Morphology of MYCN siRNA treated Kelly cells compared to control untransfected cells or irrelevant siRNA treated cells. Cells were transfected with 100 nm siRNAs then imaged 48 h later by phase contrast microscopy. A) Control untreated cells, B) cationic non‐PEG non‐targeting control siRNA, C) cationic non‐PEG MYCN siRNA, D) cationic‐PEG non‐targeting control siRNA, E) cationic‐PEG MYCN siRNA, F) anionic‐PEG non‐targeting control siRNA, and G) anionic‐PEG MYCN siRNA. H) Quantification of PI‐labeled subG1 cells after transfection with MYCN or irrelevant (IRR) control siRNAs (*n* = 2). White arrows indicate dead and dying cells; scale bars are 25 µm. DL = double‐layered.

*MYCN* silencing in neuroblastoma cells is well‐known to induce apoptosis and indeed here it induced changes in cell morphology consistent with apoptotic cell death including rounding‐up, shrinking, and lower cell density (**Figure** **4**A–G). Cell staining with propidium iodide followed by flow cytometry analysis for detection of subG1 cells is a convenient way to quantify apoptosis.^[^ ^28^ ^]^ *MYCN* siRNA transfections increased the subG1 population to more than 70%, while the PEGylated cationic and anionic formulations with irrelevant siRNA induced less than 10% subG1 cells (Figure 4H). However, irrelevant siRNAs delivered by cationic RTNs caused more than 30% apoptosis, pointing to non‐specific, cytotoxicity of the cationic formulation.

### 2.4 In Vivo Biodistribution in Mice with Neuroblastoma Xenografts

**Figure 5:**
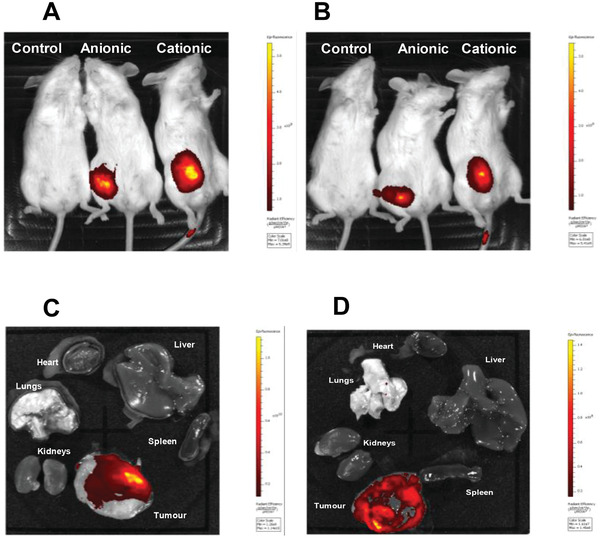
Tumor uptake after intravenous administration of cationic‐PEG and anionic‐PEG formulations. 24 h or 48 h after i.v. administration of nanocomplexes with fluorescently labeled siRNA, mice were either imaged live or culled and tumors and organs were extracted and imaged for fluorescence. Mice were imaged live A) 24 h and B) 48 h after intravenous administration of nanocomplexes containing Dy677‐labeled siRNA. Organs (heart, lung, liver, kidneys, and spleen) and tumor of a mouse that received C) anionic‐PEG nanocomplexes and D) cationic‐PEG nanocomplexes containing FAM‐labeled siRNA, 24 h after administration.

We next compared the in vivo biodistribution of nanocomplexes after intravenous delivery to mice with Kelly cell xenografts. Cationic‐PEG and anionic‐PEG, integrin targeted RTNs were prepared while cationic non‐PEGylated formulations were omitted from in vivo studies because of their size instability in serum. Live imaging of mice administered with nanocomplexes containing siRNA‐Dy677‐labeled nucleic acids showed that both cationic‐PEG and anionic‐PEG nanocomplexes (**Figure** **5**A) accumulated predominantly in tumors at 24 h after administration, which was maintained at 48 h (Figure 5B). In further experiments, to more closely interrogate the biodistribution of the nanocomplexes, mice were administered intravenously with nanocomplexes containing fluorescent, FAM‐labeled siRNA and then organs and tumors were analyzed by fluorescent microscopy 24 h later. The anionic‐PEG (Figure 5C) and the cationic‐PEG (Figure 5D) nanocomplexes both showed accumulation of fluorescence in tumors with much lower levels in other tissues (Figure 5C,D and Figure S3, Supporting Information). For example, the fluorescent radiant efficiency in tumors from anionic formulations was 4.5‐fold higher than in the lungs with none detected in other organs (Figure S3A, Supporting Information; *p* < 0.05). Although the cationic‐PEG formulations showed accumulated fluorescence in lungs, liver, and kidneys, respectively, the fluorescent radiant levels were 9.8‐, 56.3‐, and 12.5‐fold higher in tumors (Figure S3B, Supporting Information). This suggested that, while both cationic and anionic formulations accumulated in the tumors, anionic nanocomplexes displayed a higher degree of tumor specificity compared to their cationic counterparts.

**Figure 6:**
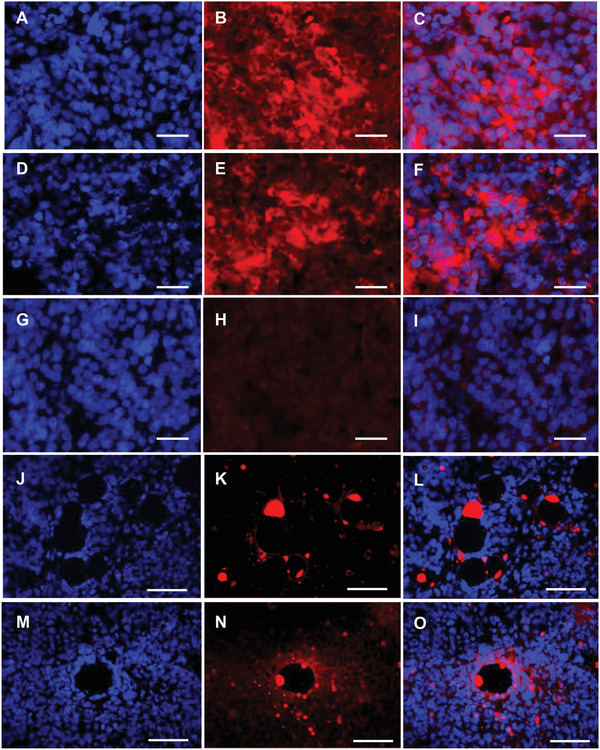
The Dy677‐siRNA fluorescence distribution was investigated in histological sections of tumors following tail‐vein injections of anionic‐PEG A–C) targeted nanocomplexes, D–F) cationic‐PEG targeted nanocomplexes, G–I) control mice, J–L) anionic‐PEG non‐targeting nanocomplexes, and M–O) cationic‐PEG non‐targeting nanocomplexes. The tumors were removed 24 h after the injection and the fluorescence was recorded. The cell nuclei were stained with DAPI (blue) and the siRNA‐Dy677 in red. DAPI staining (A,D,G,J,M), siRNA‐Dy677 (B,E,H,K,N), and merged images (C,F,I,L,O). Scale bar = 50 µm (A–I); Scale bar = 100 µm (J–O).

We then assessed whether tumor fluorescence was cell‐associated by staining tumor sections with DAPI to identify nuclei (**Figure** **6**). Here, mice were transfected with anionic‐PEG and cationic‐PEG formulations containing the integrin targeting peptide ME27, or the non‐targeting control ME72, in which the RGD, integrin‐specific motif is substituted for RGE, to assess integrin‐mediated cell targeting in vivo. Fluorescent siRNAs were abundant throughout the tumors transfected with all four formulations with no fluorescence observed in tumor sections from untreated mice (Figure 6G–I). Fluorescent siRNA was clearly located in the cytoplasm of DAPI‐stained cells after delivery with either anionic‐PEG (Figure 6A–C) or cationic‐PEG (Figure 6D–F) integrin‐targeted nanocomplexes. On the other hand, while non‐targeting, anionic‐PEG (Figure 6J–L) and non‐targeting cationic‐PEG nanocomplexes (Figure 6M–O) were present in tumors, both appeared to pool in extracellular tumor spaces rather than within cells. This suggests that after systemic administration, nanocomplex formulations first accumulate within tumors due most likely to the leakiness of the tumor neovasculature^[^ ^29^ ^]^ or endothelial transcytosis.^[^ ^12^ ^]^ Once within the tumor, targeted RTN formulations enter cells by integrin‐mediated internalization while non‐targeted formulations remain extracellular.

### 2.5 In Vivo *MYCN* Silencing in Tumor Xenografts

**Figure 7:**
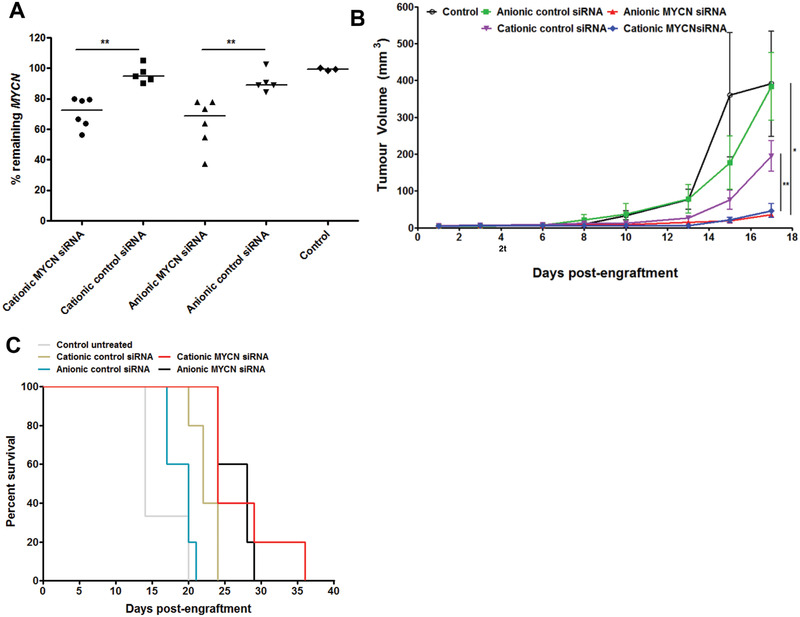
In vivo silencing of *MYCN* and effects on tumor growth following single and multiple intravenous administrations of MYCN siRNA nanocomplexes. A) qRT‐PCR analysis of *MYCN* silencing in NSG mice bearing subcutaneous Kelly tumors 48 h after intravenous administration of cationic‐PEG and anionic‐PEG nanocomplexes containing 25 μg *MYCN* siRNA (n = 6), control siRNA (n = 5) or untreated mice (n = 3). Silencing was normalized to the mean control siRNA while median values are presented by horizontal lines and asterisks indicate statistical significance (**p < 0.01). Mice were treated with cationic‐PEG and anionic formulations containing 25 µg of *MYCN* or control siRNA, delivered six times intravenously, at 48 h intervals from day 2 to day 12 in NSG mice with Kelly cell xenografts and then B) average tumor volume was determined (n = 5 for each of the five groups; *p < 0.05). C) Survival of NSG mice shown in a Kaplan–Meier plot.

We next investigated the therapeutic potential of siMYCN delivered by our nanocomplexes to mice with Kelly cell xenografts. Tumors showed *MYCN* silencing of 30% (range: 20–44%) and 36% (range: 22–63%) for cationic‐PEG and anionic‐PEG nanocomplexes, respectively, relative to control siRNA formulations (*p* < 0.01) (**Figure** **7**A). Therapeutic studies with RTNs were then conducted by six intravenous injections of siMYCN nanocomplexes given at 48 h intervals. Tumor retardation was observed for individual mice treated over time with the anionic siMYCN formulations (Figure S4A, Supporting Information) performing better than their cationic counterparts (Figure S4B, Supporting Information), compared to control siRNA groups. This was confirmed by the observation that the average tumor volumes at day 17 post‐engraftment of mice receiving siMYCN in cationic complexes (when all mice were still alive) was reduced to ≈46 mm^3^, whereas the average tumor volume in mice receiving anionic siMYCN nanocomplexes was 36 mm^3^ (*p* < 0.05), while the control siRNA group was ≈195 mm^3^ (*p* < 0.01), (Figure 7B). There was no average difference in tumor growth rates between cationic‐PEG or anionic‐PEG siMYCN nanocomplexes. The cationic control siRNA formulation appeared to slow tumor growth more than the anionic control siRNA formulations although this was not significant (Figure 7B) and did not lead to any significant effects on survival (Figure 7C). In addition, there was no significant adverse effect on body weight of mice receiving repeated doses of either the anionic or cationic RTNs, irrespective of whether they received therapeutic MYCN siRNA or control siRNA, during the whole treatment period (Figure S5A‐F, Supporting Information).

As well as reduced tumor volumes, survival was improved for mice receiving siMYCN compared to those receiving control siRNA or the untreated mice (Figure 7C). By day 20, all untreated mice were culled due to tumor sizes exceeding acceptable limits. The survival time of mice that received anionic siMYCN formulations was 38% longer than those receiving anionic control siRNA nanocomplexes, whereas mice receiving cationic siMYCN outlived the mice receiving cationic controls by 50%.

Histological sections of tumors from the last surviving mouse from each group (control untreated mouse, cationic‐PEG siMYCN, and anionic‐PEG siMYCN) were stained with hematoxylin and eosin (H&E) and examined for evidence of a therapeutic effect on the tumor such as extent of vascularization, and necrosis (Figure S6, Supporting Information). Vascularity was comparable among all groups, whereas the last surviving cationic‐PEG mouse showed more marked tumor cell death with a prominent perivascular survival effect compared with the anionic‐PEG mouse and the control untreated mouse (Figure S6, Supporting Information). Histological analysis of lung, kidney, liver, and spleen from mice injected with cationic siRNA nanocomplexes did not reveal any observable differences from organs of untreated mice (Figure S7, Supporting Information).

### 2.6 Dose Modeling

Next, we investigated the possibility of using the experimental data to establish a mathematical model to describe the effects of siMYCN nanoparticles on neuroblastoma growth using population K‐PD modeling with a simple exponential growth model chosen. There was no significant improvement in fit when anionic and cationic vehicles were separated. When analyzing the effect of the irrelevant control, fit was significantly improved (*p* < 0.001) with the irrelevant control estimated to have 29% the activity of the active. Further information is provided in Supporting Information, with model parameter estimates (Table S2, Supporting Information, and goodness‐of‐fit plots [Figure S8, Supporting Information]).

A model was successfully established using a K‐PD model as a structural submodel, while random effects for growth rate constant and baseline tumor volume were also estimated, but no covariate was identified. The model was used to evaluate the K‐PD properties difference between the two formulations of nanoparticles and no significant difference was found. Effect of the control irrelevant siRNA on neuroblastoma growth was also tested, and interestingly irrelevant siRNA appears to be not completely inert but exerts an inhibitory effect on neuroblastoma growth. SiRNAs, may display sequence dependent immunogenicity in mice facilitated by TLR7^[^ ^30^ ^]^ however, in our control siRNA (5′‐UAACGACGCGACGAACGUAATT‐3′) the known immunostimulatory motifs, for example, 5′‐UGUGU‐3′, 5′‐GUCCUUCAA‐3′, and 5′‐UGU‐3′ are missing,^[^ ^30^, ^31^ ^]^ which should reduce the risk of stimulation and so this will require further investigation.

## 3 Conclusions

The RTNs reported here showed significant *MYCN* silencing in vitro in neuroblastoma cells with little cytotoxicity. RTNs overcome a major limitation in the development of siRNA cancer therapies in that they avoid significant clearance by the liver allowing accumulation in the tumor, probably through a leaky tumor/endothelial barrier, while their integrin‐targeting and fusogenic properties enable efficient transfection of the target tumor cells. Anionic RTNs were as efficient and specific as their cationic counterparts, and, in addition, offer advantage of reduced systemic and cellular toxicity. In vivo, the RTNs enter the tumors efficiently, with little off‐target biodistribution, and transfect tumor cells in an integrin‐mediated fashion. Delivery of siMYCN resulted in retardation of tumor growth, improving survival of mice. These nanocomplexes, thus, allow for the specific targeted enhancement of nucleic acid delivery and could provide improved non‐viral vectors to deliver therapeutic cargos in a variety of disorders.

## 4 Experimental Section

### Materials

1,2‐di‐O‐octadecenyl‐3‐trimethylammonium propane (DOTMA), 1,2‐dioleoyl‐*sn*‐glycero‐3‐phospho‐(1′‐rac‐glycerol) (DOPG), 1,2‐dioleoyl‐*sn*‐glycero‐3‐phosphoethanolamine (DOPE), and 1,2‐dipalmitoyl‐sn‐glycero‐3‐phosphoethanolamine‐*N*‐[methoxy(polyethylene glycol)‐2000] (DPPE‐PEG_2000_) were purchased from Avanti Polar Lipids, Inc. (Alabaster, AL, USA). Integrin‐targeting peptide ME27 (K_16_RVRRGACRGDCLG) and non‐targeting control peptide ME72 (K_16_RVRRGACRGECLG) were synthesized by China Peptides (Shanghai, China).

siMYCNs for in vitro transfections were purchased from Eurogentec (Seraing, Liege, Belgium) with sense strands shown below;

*MYCN*‐1: 5′‐CGGAGAUGCUGCUUGAGAA‐3′

*MYCN*‐2: 5′‐CGGAGUUGGUAAAGAAUGA‐3′

*MYCN*‐3: 5′‐CAGCAGUUGCUAAAGAAAA‐3

The in vivo siRNAs, including *MYCN*‐3 and control siRNA (UGGUUUACAUGUUGUGUGA) were purchased from GE Healthcare (Amersham, UK) while Silencer Negative Control #1 (Irrelevant control siRNA 5′‐UAACGACGCGACGAACGUAATT‐3′) was purchased from Applied Biosystems (Warrington, UK). Labeled siRNAs including control siRNA‐fluorescein (siRNA‐FAM) and Dy677 control siRNA (siRNA‐Dy677) were purchased from GE Healthcare (Amersham, UK).

Lipofectamine 2000 (L2K) was purchased from Thermo Fisher (Paisley, UK) and mouse serum from Sigma‐Aldrich (Gillingham, UK).

### Liposome Preparation and Nanocomplex Formation

The lipids, including cationic DOTMA, anionic DOPG, the neutral fusogenic lipid DOPE, and PEG‐lipid DPPE‐PEG_2000_ were dissolved in chloroform to a concentration of 10 mg mL^−1^. Liposomes were prepared by preparing mixtures of lipids at the following molar ratios: DOTMA:DOPE (50:50), DOTMA:DOPE:DPPE‐PEG_2000_ (49.5:49.5:1), and DOPG:DOPE:DPPE‐PEG_2000_ (49.5:49.5:1). The chloroform was evaporated in a rotary evaporator (BÜCHI Labortechnik AG, Flawil, Switzerland) and the lipid film was hydrated in water followed by sonication at RT for 45 min to generate liposomes as described previously.^[^ ^32^ ^]^

Cationic, non‐PEGylated (cationic non‐PEG) nanocomplexes were formulated by mixing DOTMA/DOPE liposomes with peptides and siRNA at a weight ratio of 1:4:1, liposome:peptide:siRNA and in that order of mixing, with rapid mixing on addition of siRNA, followed by incubation for 30 min at RT to allow for complex formation. Cationic‐PEG nanocomplexes were prepared in a similar way but with DOTMA/DOPE/DPPE‐PEG_2000_ liposome instead of DOTMA/DOPE. The anionic‐PEGylated nanocomplex formulations were prepared in a double‐layered approach by first preparing a cationic non‐PEG nanocomplex formulation at a weight ratio of 0.75:3:1 (DOTMA/DOPE:liposome:peptide:siRNA), and then adding anionic‐PEG liposomes (DOPG:DOPE:DPPE‐PEG_2000_) at a weight ratio of 19:1 (anionic‐PEG liposome:siRNA). The mixture was incubated at RT for 30 min then diluted for use as required. Lipofectamine 2000 (L2K) siRNA complexes were prepared by mixing with siRNA at a weight ratio of 4:1 (L2K:siRNA) followed by incubation at RT for 20 min before use.

### Particle Sizing and Zeta Potential Measurement

Size and surface charge (ζ potential) measurements were performed with a Malvern Nano ZS zetasizer (Malvern, UK). Nanocomplexes were prepared (5 µg mL^−1^ with respect to siRNA) then diluted with deionized water or PBS to a final volume of 1 mL. Measurements were performed in the NanoZS at a temperature of 25 °C. DTS version 5.03 software provided by the manufacturer was used for data processing. Nanoparticle sizes and particle concentration were also determined by NTA using a NanoSight LM20 (NanoSight, Amesbury, UK) equipped with a sample chamber with a 405 nm blue laser and a Viton fluoroelastomer O‐ring.^[^ ^33^ ^]^ Nanocomplexes were prepared at 10 µg siRNA in 100 µL water and then diluted with PBS before measurement as required. NTA measurements were performed at 21 °C. Each experiment was repeated at least thrice and the results were presented as mean hydrodynamic sizes ± S.D.^[^ ^33^ ^]^

### Turbidity Assay

The absorbance of complexes in the absence and presence of mouse serum at a range of concentrations (0–50% v/v) was measured at 500 nm using a FLUOstar Optima spectrophotometer (BMG Labtech, Aylesbury, UK) as described previously,^[^ ^14e^ ^]^ with a corresponding amount of serum used as a reference.

### Heparin Dissociation Assay

Briefly, 0.2 µg siRNA was mixed with PicoGreen reagent (1:150) (Invitrogen, Paisley, UK) at RT in 10 mm Tris‐HCl, 1 mm EDTA, and pH 7.5 (TE) buffer, and the siRNA/PicoGreen mixture was then formulated into nanocomplexes as described above. Heparin sulfate (Sigma, Poole, UK) was added to the complexes formulated with PicoGreen in a range of concentrations (0.05–1 U mL^−1^) and then fluorescence (480 nm excitation peak and 520 nm emission peak) was measured in an OPTIMA Fluostar. In each experiment, naked siRNA stained with PicoGreen was used to normalize the PicoGreen signal detected from the complexes.

### Transmission Electron Microscopy

Nanocomplexes were prepared as described above and were applied onto a glow‐discharged 300‐mesh copper grid coated with a Formvar/carbon support film (Agar Scientific). The grid was dried by blotting with filter paper then negatively stained with 1% w/v uranyl acetate before blotting with filter paper and air‐dried. Imaging was performed under a Philips CM120 BioTwin TEM, operated at an accelerating voltage of 120 KV.

### Cell Culture

Human neuroblastoma cell lines Kelly and LAN‐5 were obtained from the American Type Culture Collection (ATCC, Teddington, UK) and certified as mycoplasma free. Kelly cells were cultured in RPMI1640+GlutaMAX (Invitrogen, Paisley, UK) supplemented with FBS (10%), HEPES (25 mm), and penicillin/streptomycin (100 U mL^−1^). LAN‐5 cells were cultured in MEM with FBS (10%), L‐glutamine (2 mm), and penicillin/streptomycin (100 U mL^−1^). All cells were maintained in a humidified atmosphere of air (95%) and CO_2_ (5%) at 37 °C.

### siRNA Transfection

Kelly or LAN‐5 cells were seeded (3 × 10^5^ cells per well) in 12‐well plates and then the following day when 50–60% confluent, nanocomplexes were prepared as above in OptiMEM (Section: Liposome Preparation and Nanocomplex Formation), containing siMYCN or control siRNA (100 nm), and transfection incubations were performed for 4 h. The transfection medium was replaced with complete media and the cells were incubated at 37 °C for a further 48 h. Cells were then harvested by trypsinization and homogenized with Qiagen shredders (Qiagen, Crawley, UK). Total RNA was extracted from the homogenate using the RNeasy Kit (Qiagen, Crawley, UK) and each sample treated with DNase (Invitrogen, Paisley, UK) to eliminate any contaminating DNA. RNA samples were stored at −80 °C.

### Quantitative Real‐Time PCR

Total RNA (200 ng per reaction) was used in a one‐step quantitative real‐time PCR (qRT‐PCR) (SensiFast Probe Hi‐Rox One‐Step Kit; Bioline, London, UK) that combines the reverse transcription step with the quantitative PCR (qPCR) reaction. Human *MYCN* and *ACTB* (encoding β‐actin), were quantified by Taqman primers and probes (Hs00232074_m1 and Hs99999903_m1 for *MYCN* and *ACTB*, respectively; Thermo Fisher Scientific, Northumberland, UK). The qRT‐PCR assays were performed in a Bio‐Rad CFX96 Real‐Time PCR Detection System with the following parameters: 45 °C for 20 min, 95 °C for 2 min, and then 40 cycles at 95 °C for 15 s and 60 °C for 1 min. Relative expression levels were calculated using the delta‐delta Ct (2^−ΔΔCt^) method.^[^ ^34^ ^]^

### Cell Viability Assay

Cell viability was assessed in 96‐well plates using the CellTiter 96 Aqueous One Solution Cell Proliferation Assay (Promega, Southampton, UK). Kelly or LAN‐5 cells were transfected as above, then after 24 h the medium was substituted for a growth medium containing 20 µL of CellTiter 96 Aqueous One Solution reagent, incubated for 2 h, then absorbance at 490 nm measured with a FLUOstar Optima (BMG Labtech, Aylesbury, UK). Cell viability for each complex was expressed as a percentage of the viability of control untreated cells. Cell proliferation analysis following siRNA transfections was performed 48 h later.

### Propidium Iodide Staining for Sub‐G1 DNA Content

Kelly cells were transfected with nanocomplexes then trypsinized at 24 h and fixed in ice‐cold 70% ethanol for 30 min, rinsed twice in phosphate‐citrate buffer (0.2 m Na_2_HPO_4_/0.1 m citric acid, pH 7.8) by centrifugation at 500 × *g*, and resuspended in propidium iodide (200 µL of 50 µg mL^−1^) in PBS and RNaseA solution (50 µL of 100 µg mL^−1^ in distilled water). The cells were analyzed with a BD LSRII flow cytometer (BD Biosciences, Wokingham, UK). A maximum of 10 000 events were collected per sample then data analyzed using Flo‐jo V8 software.

### Complement Activation Assays

Characterization and functional assessments of complement pathways were performed as described previously^[^ ^35^ ^]^ by measuring increases in the serum complement‐activation products, C5a and sC5b‐9, by enzyme linked immunosorbent assays (ELISA kits, Quidel, San Diego, USA). Complement activation was initiated by adding the appropriate quantities of nanocomplex (in 10 µL) to undiluted human serum (40 µL) in Eppendorf tubes in a shaking water bath at 37 °C for 30 min. Reactions were terminated by the addition of ice‐cold sample‐diluent, from the assay kit, containing EDTA (25 mm). Nanocomplexes were removed by centrifugation, and complement activation products were measured by ELISA. Control serum incubations contained buffers used for liposome suspension. Zymosan (0.2 mg mL^−1^) was prepared as described before^[^ ^35^ ^]^ and was used as a positive control for generating C5a and sC5b‐9.

### In Vivo Delivery to Xenograft Tumor Models

All animal procedures were approved by UCL animal care policies and were performed under Home Office Licenses, issued in accordance with the United Kingdom Animals (Scientific Procedures) Act 1986 (UK). 6 to 8 weeks old, NOD‐SCID gamma (NSG) mice of mixed gender, (Charles River, Margate, UK), were injected subcutaneously in the right posterior flank with 3 × 10^6^ human neuroblastoma Kelly cells. After ≈2 weeks, tumors reached 8–10 mm in size, at which point cationic or anionic RTN complexes (100 µL in 5% D‐glucose) containing siRNA‐FAM or siRNA‐Dy677 (16 µg), were injected intravenously in the tail. The nanocomplexes were made as described above (Section: Liposome Preparation and Nanocomplex Formation) except that the final added components (siRNA for cationic RTNs; anionic‐PEG liposome in anionic‐PEG RTNs) were mixed with nuclease‐free water to make a volume of 90 µL per sample. Following the final incubation, 10 µL of 50% D‐glucose were added to make the final volume of 100 µL per injection and the RTNs were stored at 2–8 °C until needed. 24 or 48 h after injection, mice were either imaged live, or killed and tumors and organs resected and imaged using an IVIS Lumina Series III imaging system (Perkin Elmer, Seer Green, UK). Images were processed with the Living Image software (Perkin Elmer, Seer Green, UK).

For silencing experiments, ≈2 weeks after Kelly cells were xenografted, the mice were injected by tail vein with 100 µL of cationic or anionic RTN complexes containing 25 µg siMYCN or control siRNA in 5% D‐glucose. 48 h later the mice were culled and the tumors were collected in RNAlater (Invitrogen, Paisley, UK) and homogenized in lysis buffer with a Precellys24 tissue homogenizer (Stretton Scientific, Stretton, Derbyshire, UK) and then centrifuged at 14170 × *g* for 10 min at 4 °C. The supernatant was removed and centrifuged for a further 10 min at 4 °C. Total RNA was extracted from lysates using the RNeasy kit (Qiagen, Crawley, UK) and the qRT‐PCR assay was performed as described above.

For tumor progression experiments, the RTN injections commenced when tumors were palpable. Mice received six consecutive injections of nanocomplexes containing 25 µg of siMYCN or control siRNA administered from day 2 post‐engraftment (days: 2, 4, 6, 8, 10, and 12) and tumor progression was monitored as described previously.^[^ ^36^ ^]^ The tumors from the last surviving mouse of each group (cationic and anionic siMYCN formulations, and untreated control) were then placed in paraformaldehyde (PFA;4% w/v) (Sigma‐Aldrich, Gillingham, UK) for 3 h and embedded in paraffin wax and 7 µm sections mounted on polylysine‐coated slides. For histological analysis, sections were serially rehydrated and stained with H&E, followed by serial dehydration with ethanol and mounted with glass coverslips.

### Preparation of Frozen Tissue Sections

Freshly dissected tumors from mice transfected with nanocomplexes containing siRNA‐Dy677 were placed onto prelabeled tissue base molds. The tissue block was covered with cryo‐embedding media OCT (Leica microsystems, Milton Keynes, UK). Base mold containing tissue block was snap frozen in isopentane (VWR International, Lutterworth, UK), that had been prechilled in liquid nitrogen, and then transferred to a cryotome cryostat, precooled to −20 °C. 10 µm tissue sections were prepared using a cryotome and mounted on Superfrost Plus glass slides (Fisher Scientific UK, Loughborough, UK). The sections were dried at RT and then stored at −80 °C until required.

### Staining of Frozen Sections

Tissue sections were rinsed in PBS briefly to remove any media components, then fixed in precooled (−20 °C) acetone for 10–15 min and rinsed with three changes of PBS, 5 min each. Tissue sections were stained with DAPI for 15 min at RT in the dark, then washed with three further changes of PBS for 5 min each. Sections were mounted using ProLong Gold antifade mountant (Thermo Fisher Scientific, Hemel Hempstead, UK). Micrographs were taken using Leica upright fluorescence (Leica DFC310 FX) at 200× magnification.

### Histological Analysis of Toxicity

NSG mice were injected by tail vein with 20 µg siRNA‐Dy677 in cationic‐PEG nanocomplexes and were sacrificed 24 h later. Untransfected mice were used as controls. Tissue samples from lung, liver, kidney, and spleen were fixed in 4% PFA and 70% ethanol. Then the organs were transferred to neutral buffered formalin solution (Genta Medical, York, UK) prior to 13 h of overnight processing on a Leica PELORIS II Tissue Processor. In brief, over multiple incubations, samples were dehydrated in 99% industrial denatured alcohol/99% industrial methylated spirit (Genta Medical, York, UK), cleared in xylene (Genta Medical, York, UK), and impregnated with CellWax Plus [S] paraffin wax (Cell Path, Newtown, Powys, UK). Samples were embedded using Sakura Tissue‐Tek TEC 5 console system. Formalin‐fixed‐paraffin‐embedded sections were generated at 3 µm thickness on a Thermo Scientific Shandon Finesse ME+ microtome. Sections were stained on the Leica Autostainer XL ST5010, an automated H&E workstation, and mounted using LEICA CV5030 automated cover slipper machine. The stained H&E sections were scanned on the Leica Aperio CS2 Scanner at 40× objective.

### Dose Modeling

A pharmacokinetic/pharmacodynamic model was developed of changes in tumor volume over time with details provided in the Supporting Information.

### Statistics

The data presented in this study were expressed as the mean ± standard deviation and were analyzed using a two‐tailed, unpaired Student's *t*‐test or one‐way analysis of variance and Bonferroni's post hoc analysis, where applicable. Non‐parametric data were analyzed using a Mann–Whitney U test.

## Conflict of Interest

A.D.T. is a consultant for Nanogenics Ltd. S.L.H. holds equity in Nanogenics Ltd.

## Supporting information

Supporting Information

## Data Availability

Data available on request from the authors.
