# Supplementary material for: Integrin‐Targeted, Short Interfering RNA Nanocomplexes for Neuroblastoma Tumor‐Specific Delivery Achieve MYCN Silencing with Improved Survival
Source: Adv Funct Mater. 2021 Jun 30;31(37):2104843. doi: 10.1002/adfm.202104843 (PMC9178728; doi:10.1002/adfm.202104843)
Supplement: Supplementary file 1 — Supporting Information [file ADFM-31-0-s001.pdf]

© 2021 Wiley-VCH GmbH

ADVANCED FUNCTIONAL MATERIALS

Supporting Information

for *Adv. Funct. Mater.,* DOI: 10.1002/adfm.202104843

Integrin-Targeted, Short Interfering RNA Nanocomplexes for Neuroblastoma Tumor-Specific Delivery Achieve *MYCN* Silencing with Improved Survival

*Aristides D. Tagalakis, Vignesh Jayarajan, Ruhina Maeshima, Kin H. Ho, Farhatullah Syed, Lin-Ping Wu, Ahmad M. Aldossary, Mustafa M. Munye, Talisa Mistry, Olumide Kayode Ogunbiyi, Arturo Sala, Joseph F. Standing, Seyed M. Moghimi, Andrew W. Stoker, and Stephen L. Hart**

Supplementary Information on PK/PD Modelling of Tumor Growth

Materials and Methods

Tumor volume with time for each individual was plotted and the following candidate growth models considered: exponential, logistic, Gompertz and generalized logistic (Ribba et al. 2014). Since no measurements of MYCN siRNA were made, a compartment with an apparent exponential decay in effect was added. Inhibition of growth was then estimated to be a function of this according to the classical Hill equation. The differential equations for the exponential growth model were therefore:

$$\frac{d}{dt}X = -kX,$$

$$\frac{d}{dt}T = a\left(1 - \frac{E_m X}{E_{50} + X}\right)T,$$

where X was the apparent proportion of MYCN siRNA remaining with time (t), and k the effect decay constant. T was the tumor volume, a the growth rate constant, E_m the maximum MYCN siRNA effect, and E_{50} the effect at the MYCN siRNA level to give half the maximum possible effect. The initial condition for the MYCN siRNA compartment was set to zero for $X(t = 0)$, with 25μg (the dose of MYCN siRNA used) added at each dosing event. The tumor volume compartment was initialised with an estimated baseline tumor volume parameter (b) for $T(t = 0)$.

Models were fitted to all samples from all mice (receiving no injection, irrelevant control siRNA, and active MYCN siRNA) simultaneously using nonlinear mixed effects with NONMEM version 7.3, using the first order conditional estimation algorithm and ADVAN6 differential equation solver (Beal et al. (2013)). Inter-individual variability was tested for all parameters using an assumed log-Normal distribution. The residual error included additive and proportional terms.

Investigation of whether the irrelevant control has some anti-tumor effect was estimated by multiplying the 25μg doses in the sytem by a parameter f, a fraction of the full MYCN siRNA effect. Whether addition of this parameter significantly improved model fit was evaluated with the likelihood ratio test, the difference in -2 log likelihood $(-2LL)$ of the models with and without the parameter being asymptotically χ_1^2 distributed. In a similar way, whether the MYCN siRNA was different with anionic or cationic vehicle was estimated by multiplying k, E_m or E_{50} by $(1 + \theta_v)$ where θ_v was the vehicle effect allowed to take values of ≥ -1. Non-nested structural models were compared using Akaike Information Criteria (AIC), where: $AIC = -2LL + 2p$ with p being the difference in number of

parameters between models. Further model evaluation consisted of plotting predictions versus observations, and standardised residuals versus time and predictions, in addition to a visual predictive check (comparison of model simulations with observed tumor volumes).

Results

The simple exponential growth model was chosen, with no improvement in AIC being found with Gompertz model (AIC 925.4 vs 931.1). there was a slight improvement in fit with the logistic model (AIC=923.3) but parameter estimation became unstable with the generalized logistic model. The maximum tumor volume in the logistic model was an unphysiologically high (115000 mm^2) and no visible improvement in goodness-of-fit plots was apparent. There was no significant improvement in fit when anionic and cationic vehicles were separated. When analyzing the effect of the irrelevant control, fit was significantly improved ($p<0.001$) with the irrelevant control estimated to have 29% the activity of the active. Model parameter estimates are given in Table S2 and goodness-of-fit plots in Figure S8

Discussion

A nonlinear mixed effects model of tumor volume with time has been developed, which describes and simulates data well (see Figure XYZ). The major findings from the modelling were that the rate constant of the effect degradation was characterized, the effect of the irrelevant siRNA control was quantified, and no significant effect of vehicle (anionic or cationic) was found. The degradation rate constant of effect was estimated to be 8.8 $days^{-1}$, which translates to an effect half-life of around 2 hours.

The irrelevant control was found to have around 29% of the activity of the active MYCN siRNA.

References

Beal, S, L B Sheiner, A Boeckmann, and R J Bauer. 2013. “NONMEM User’s Guides. (1989-2013).” *Icon Development Solutions, Ellicott City, MD, USA*.

Ribba, B., N. H. Holford, P. Magni, I. Troconiz, I. Gueorguieva, P. Girard, C. Sarr, M. Elishmereni, C. Kloft, and L. E. Friberg. 2014. “A review of mixed-effects models of tumor growth and effects of anticancer drug treatment used in population analysis.” *CPT Pharmacometrics Syst Pharmacol* 3 (May): e113.

A

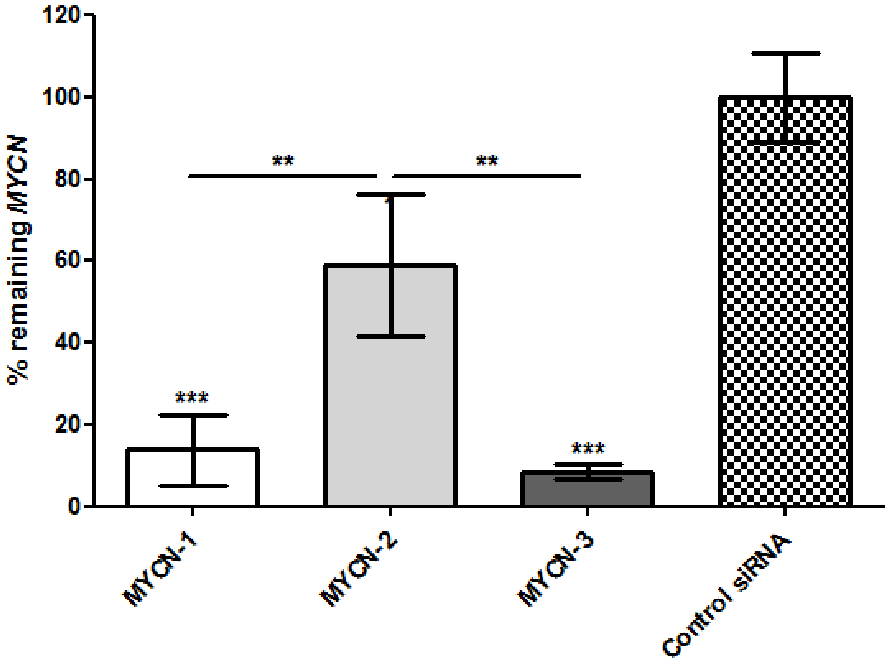

B

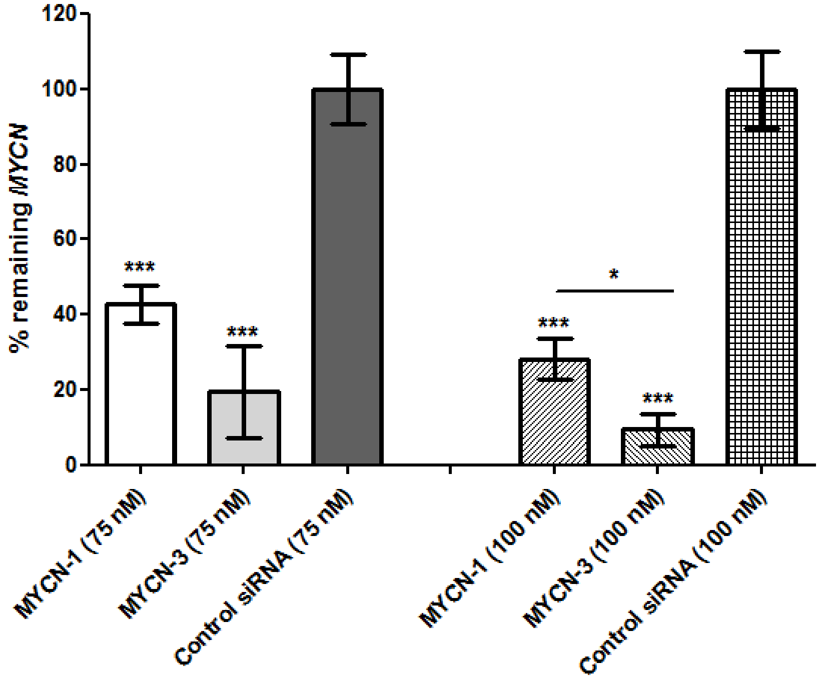

Figure S1. *MYCN* gene silencing of Kelly cells. (A) L2K formulations containing 3 different MYCN-targeting siRNAs (MYCN 1-3) were used to transfect Kelly cells at 100 nM. (B) Cationic non-PEG formulations containing peptide ME27 and 2 different MYCN-targeting siRNAs (MYCN-1 and MYCN-3) were used to transfect Kelly cells at 75 or 100 nM. All results shown (A-B) have been normalized with values for the same formulations using irrelevant control siRNA which were set at 100%. Asterisks indicate comparisons of formulations compared with the cells treated with the control siRNA or comparisons between specific formulations with statistical significance (*, $p < 0.05$; **, $p < 0.01$; ***, $p < 0.001$).

Anionic PEG DL

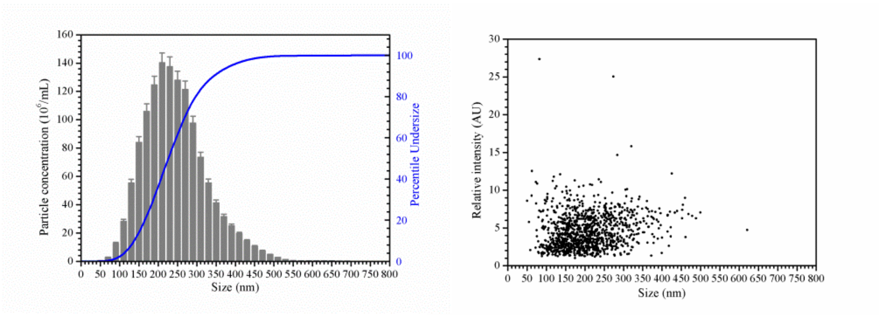

Cationic PEG

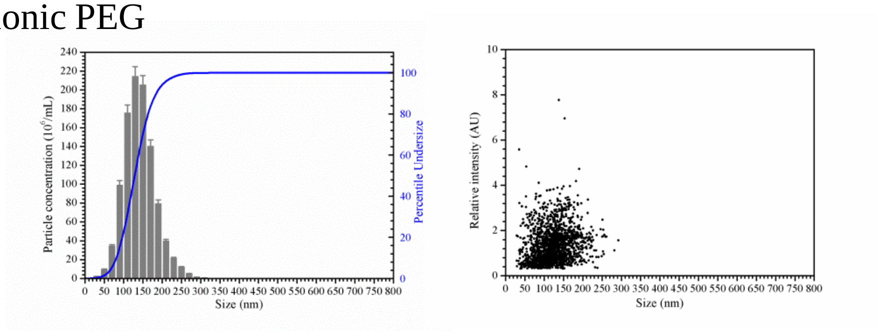

Figure S2. Characteristics of nanocomplex formulations. Typical size distribution profile (left), and 2D plots of relative light scattering intensity of particles versus the estimate of the particle size (right). DL= double-layered.

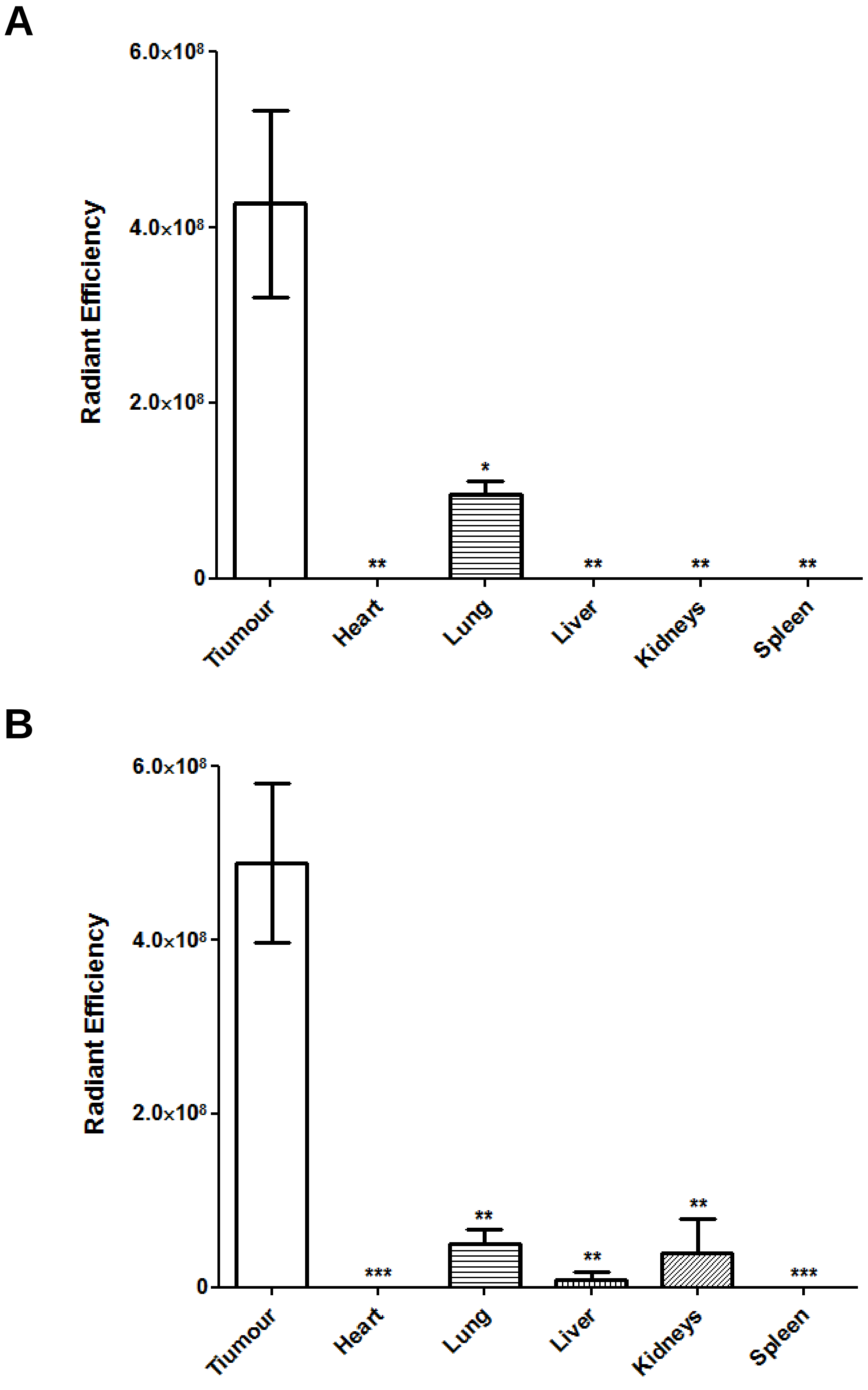

Figure S3. Radiant efficiencies (photons s^{-1} cm^{-2} $steradian^{-1}$ per μW cm^{-2}) of organs/tissues following intravenous administration of (A) anionic PEGylated or (B) cationic PEGylated nanocomplexes. 24 hours later the mice were culled and the organs and tumors excised and imaged with the IVIS III system. The uptake of siRNA-FAM was significantly more in the tumors when compared to the other organs and especially to the lungs. Asterisks indicate comparisons of the tumors with the other organs with statistical significance (*, $p < 0.05$; **, $p < 0.01$; ***, $p < 0.001$). Radiant efficiencies were measured using a Living Image 4.0 software package.

A

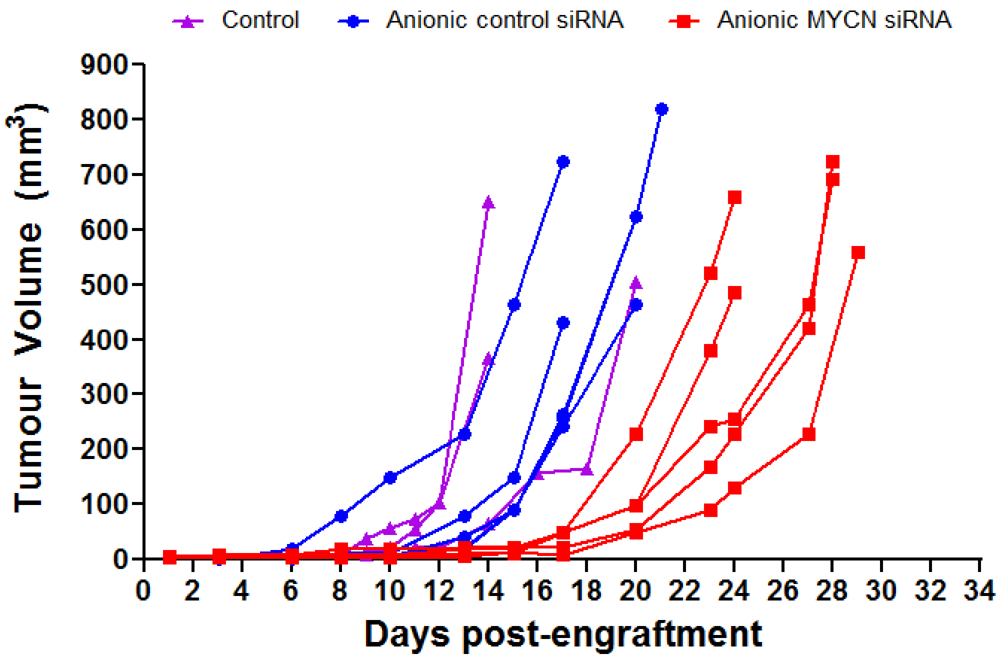

B

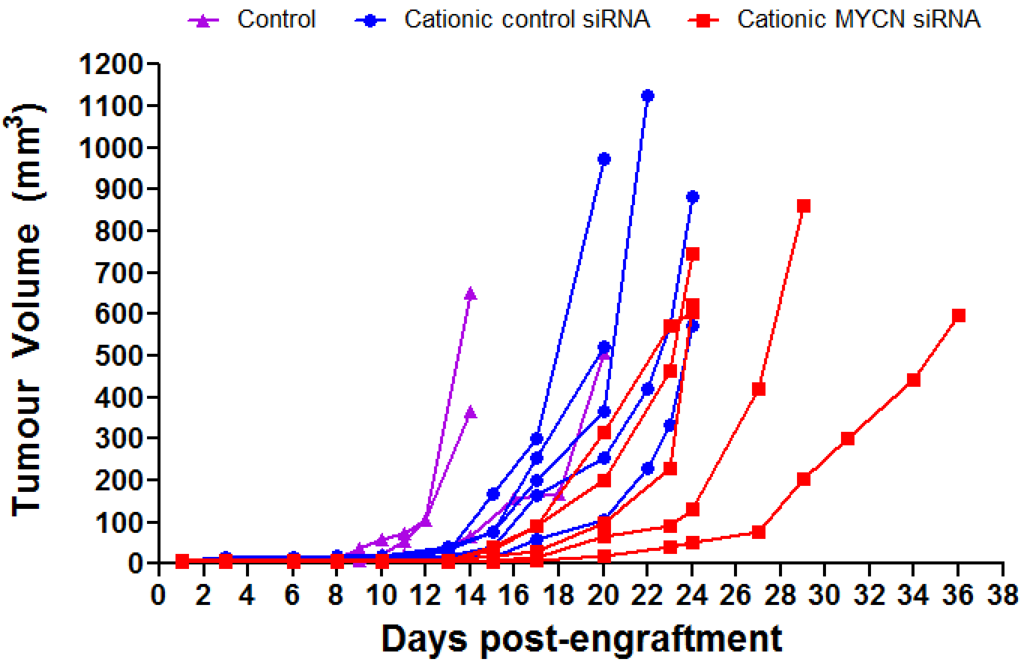

Figure S4. Growth of tumours after treatments delivered six times, at 48 h intervals from day 2 to day 12 after subcutaneous engraftment of Kelly cells. Tumor growth of each NSG mouse is represented by a single line after treatment with A) anionic PEG DL MYCN siRNA, anionic PEG DL control siRNA and B) cationic PEG MYCN siRNA and cationic PEG control siRNA. The same control untreated group is used in both A and B.

A

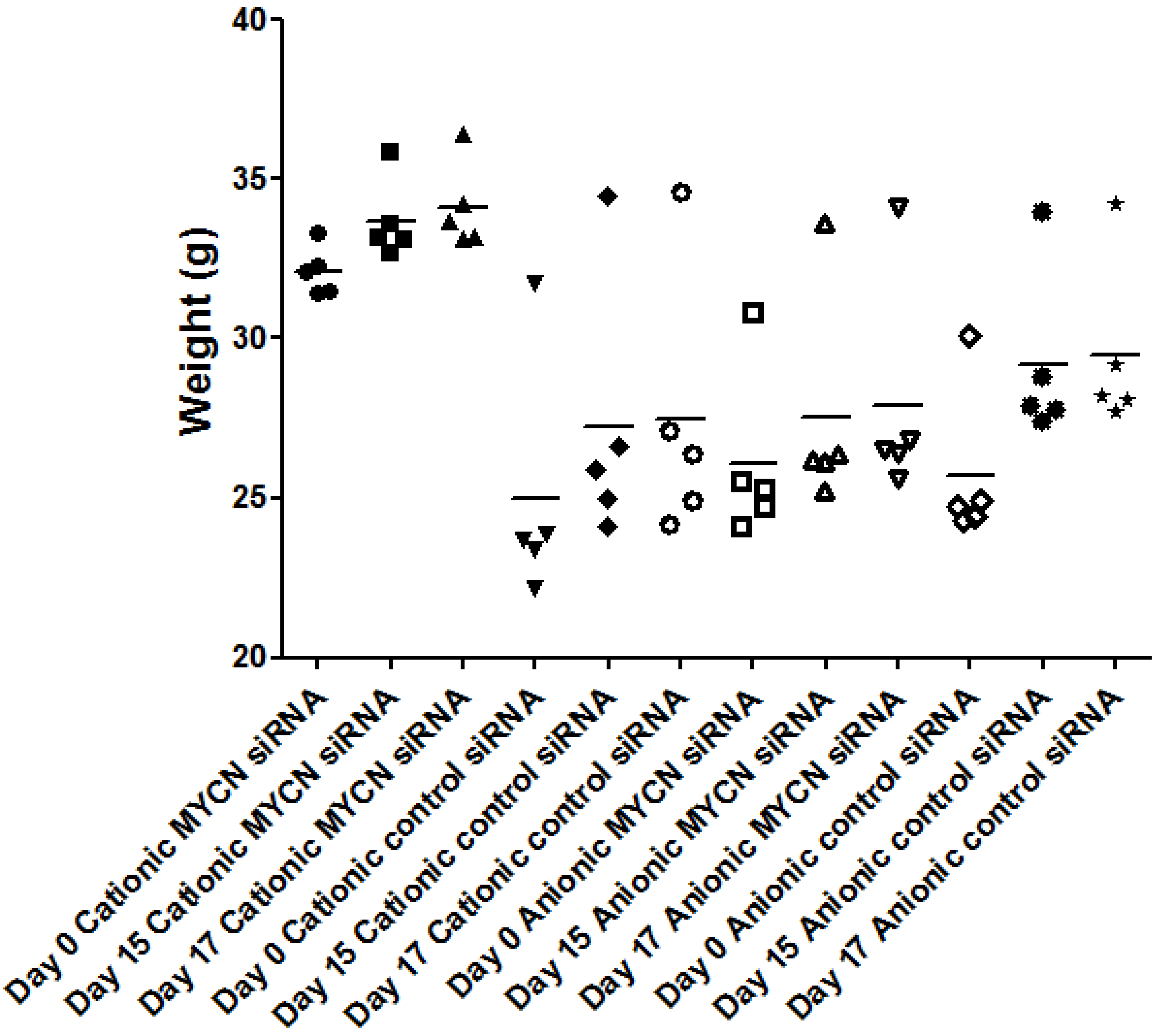

Figure S5. Weights of mice (in grams) following 6 intravenous administrations from days 2 to 12 (every 48 h) **(A)**. Mice weights receiving each nanoparticle during the experiment are shown **(B-F).** Cationic MYCN siRNA nanoparticles **(B),** Cationic non-targeting control siRNA nanoparticles **(C),** Anionic MYCN siRNA nanoparticles **(D),** anionic non-targeting control siRNA nanoparticles **(E)** and untreated mice (not injected) **(F).** Each individual mouse is represented by a symbol. The lines are the medians of the weights for each group of mice. Each individual mouse is represented by a symbol. The lines are the means of the weights for each group of mice.

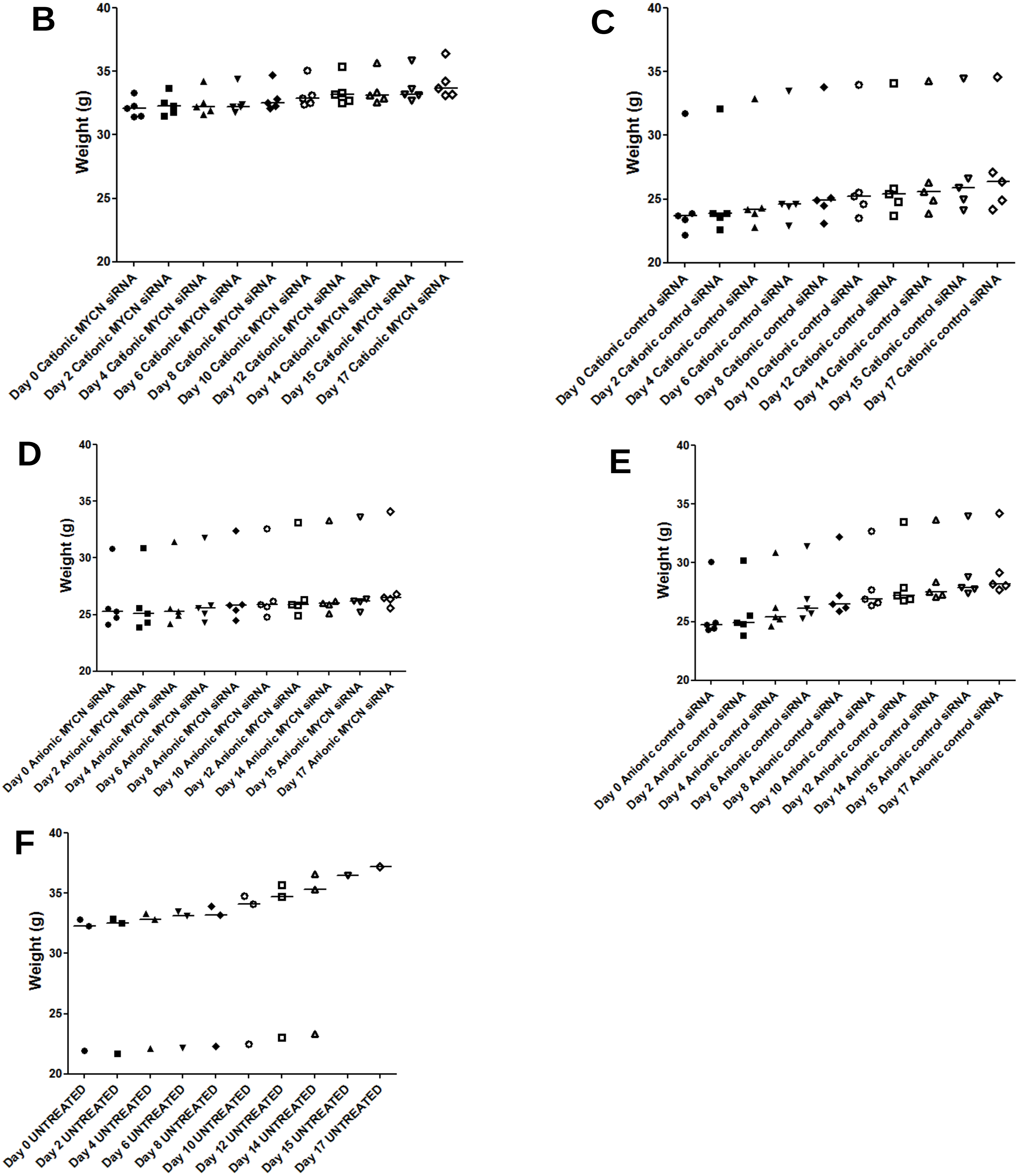
B
Weight (g)
20
25
30
35
40
Day 0 Cationic MYCN siRNA
Day 2 Cationic MYCN siRNA
Day 4 Cationic MYCN siRNA
Day 6 Cationic MYCN siRNA
Day 8 Cationic MYCN siRNA
Day 10 Cationic MYCN siRNA
Day 12 Cationic MYCN siRNA
Day 14 Cationic MYCN siRNA
Day 15 Cationic MYCN siRNA
Day 17 Cationic MYCN siRNA
C
Weight (g)
20
25
30
35
40
Day 0 Cationic control siRNA
Day 2 Cationic control siRNA
Day 4 Cationic control siRNA
Day 6 Cationic control siRNA
Day 8 Cationic control siRNA
Day 10 Cationic control siRNA
Day 12 Cationic control siRNA
Day 14 Cationic control siRNA
Day 15 Cationic control siRNA
Day 17 Cationic control siRNA
D
Weight (g)
20
25
30
35
40
Day 0 Anionic MYCN siRNA
Day 2 Anionic MYCN siRNA
Day 4 Anionic MYCN siRNA
Day 6 Anionic MYCN siRNA
Day 8 Anionic MYCN siRNA
Day 10 Anionic MYCN siRNA
Day 12 Anionic MYCN siRNA
Day 14 Anionic MYCN siRNA
Day 15 Anionic MYCN siRNA
Day 17 Anionic MYCN siRNA
E
Weight (g)
20
25
30
35
40
Day 0 Anionic control siRNA
Day 2 Anionic control siRNA
Day 4 Anionic control siRNA
Day 6 Anionic control siRNA
Day 8 Anionic control siRNA
Day 10 Anionic control siRNA
Day 12 Anionic control siRNA
Day 14 Anionic control siRNA
Day 15 Anionic control siRNA
Day 17 Anionic control siRNA
F
Weight (g)
20
25
30
35
40
Day 0 UNTREATED
Day 2 UNTREATED
Day 4 UNTREATED
Day 6 UNTREATED
Day 8 UNTREATED
Day 10 UNTREATED
Day 12 UNTREATED
Day 14 UNTREATED
Day 15 UNTREATED
Day 17 UNTREATED

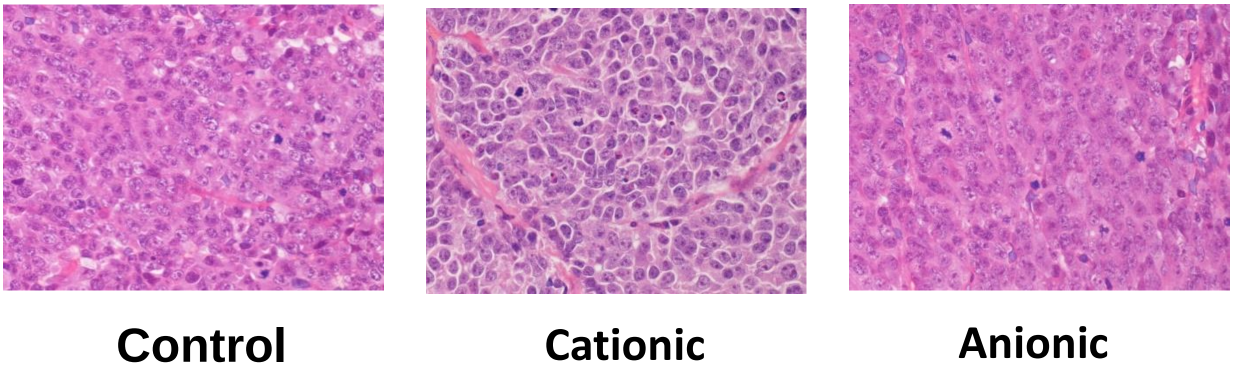

Figure S6. Tumor histology following Hematoxylin and Eosin staining. NSG mice bearing subcutaneous tumors were treated with cationic PEG MYCN siRNA or anionic PEG siRNA nanocomplexes on days 2, 4, 6, 8, 10 and 12 post-engraftment or were left untreated. Representative sections are shown of the viable tumors of the last surviving mouse of each group. Magnification is *x40* for tumors.

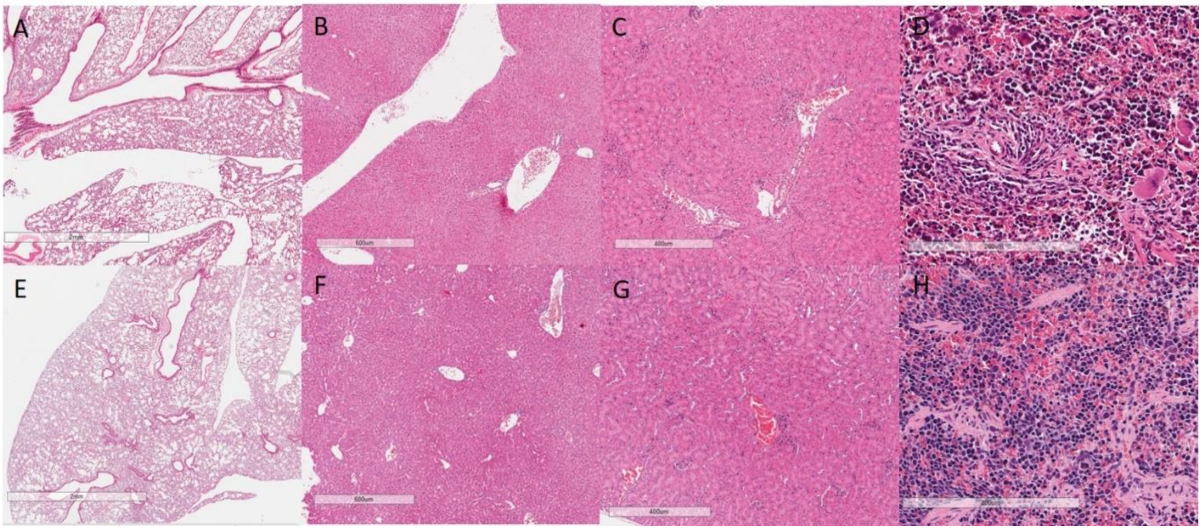

Figure S7. H&E staining of main organs from SCID mice 24 h after intravenous administration of cationic nanocomplex containing Dy677-labelled siRNA. A) lung, B) liver, C) kidney and D) spleen from an untransfected mouse. E) lung, F) liver, G) kidney and H) spleen from an transfected mouse. Three SCID mice were used and no major change was seen among the four organs compared with the untransfected mouse. The scale bars represent 2 mm (A and E), 600 µm (B and F), 400 µm (C and G), and 200 µm (D and H) respectively.

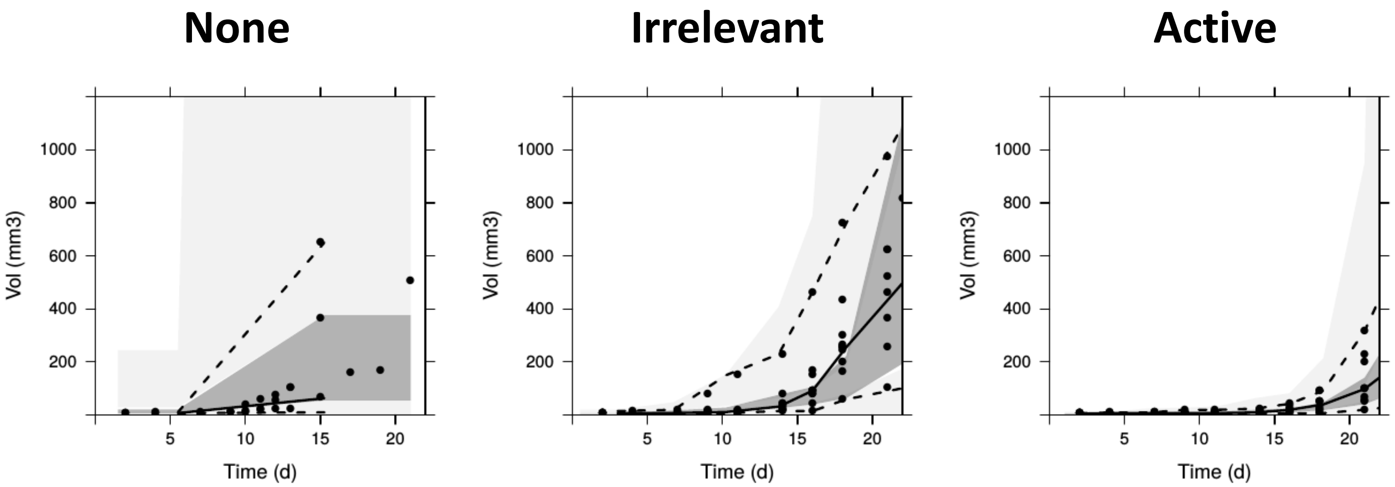

Figure S8. Visual predictive checks for: a. No treatment; b. Irrelevant control siRNA; c. Active MYCN siRNA. Points are observed tumor volume (mm^3), solid lines observed median, dark shaded area represents the 95% CI of the model simulated median, dashed lines observed the 5th and 95th percentiles, and the light shaded areas the 95% CI of the 5th and 95th percentiles.

Table S1: Size and concentration of nanoparticles by NTA.

**Sample name**	**Size** (nm)	**Ave particle conc.** (10^8/mL)	**siRNA molecules per particle**
Anionic-PEG	243	13,986	3,120
Cationic-PEG	144	8,516	5,120

Table S2: Nonlinear mixed effects model parameter estimates.

**Parameter**	**Estimate (%RSE)**	**IIV %CV (%RSE)**
k (/d)	16.17(237.8)	-
b (mm^3)	2.02(5.7)	0.01(99.4)
a (/d)	0.33(3.9)	0.04(33.2)
E_m (mcg/d)	11.07(204.6)	-
E_{50} (mcg)	2.48(83)	-
f	0.29(38.3)	-
$\sigma_{(prop\ error)}$	0.19(13.9)	-
$\sigma_{(add\ error)}$ (mm^3)	0.04(391)	-
